# Effects of three long-acting reversible contraceptive methods on HIV target cells in the human uterine cervix and peripheral blood

**DOI:** 10.1186/s12958-019-0469-8

**Published:** 2019-02-22

**Authors:** Liping Li, Jie Zhou, Weijia Wang, Lina Huang, Jiaoqin Tu, Lyndsey Baiamonte, Moselle Stark, Mistie Mills, Thomas J. Hope, Erma Z. Drobnis, Alison J. Quayle, Danny J. Schust

**Affiliations:** 1 Department of Obstetrics and Gynecology, Guangzhou First People’s Hospital, South China University of Technology School of Medicine, Guangzhou, China; 2 Department of Obstetrics, Gynecology and Women’s Health, University of Missouri School of Medicine, Columbia, MO USA; 3 Department of Microbiology, Immunology and Parasitology, Louisiana State University Health Sciences Center, New Orleans, Louisiana USA; 4 Department of Cell and Molecular Biology, Northwestern University Feinberg School of Medicine, Chicago, IL USA

**Keywords:** Hormonal contraception, HIV, CXCR4, CCR5, Depot medroxyprogesterone acetate (DMPA)

## Abstract

**Background:**

Hormonal contraceptives, particularly depot medroxyprogesterone acetate (DMPA), have been reported to be associated with substantially enhanced HIV acquisition; however, the biological mechanisms of this risk remain poorly understood. We aimed to investigate the effects of different hormonal contraceptives on the expression of the HIV co-receptors, CXCR4 and CCR5, on female endocervical and peripheral blood T cells.

**Methods:**

A total of 59 HIV-negative women were enrolled, including 15 initiating DMPA, 28 initiating a levonorgestrel-releasing intrauterine device (LNG-IUD) and 16 initiating an etonogestrel (ETG)-delivering vaginal ring. Peripheral blood and endocervical cytobrush specimens were collected at enrollment and 3–4 weeks after contraception initiation to analyze the expression of CXCR4 and CCR5, on CD4^+^ and CD8^+^ T cells using flow cytometry.

**Results:**

Administration of DMPA increased the percentages of CD4^+^ and CD8^+^ T cells expressing CCR5 in the endocervix but not in the peripheral blood. Administration of the LNG-IUD or the ETG vaginal ring did not affect the percentages of T lymphocytes expressing CXCR4 or CCR5 in the female cervix or peripheral blood.

**Conclusions:**

Increase in the percentage of endocervical T cells expressing CCR5 upon DMPA exposure provides a plausible biological explanation for the association between DMPA use and an elevated risk of HIV infection.

**Electronic supplementary material:**

The online version of this article (10.1186/s12958-019-0469-8) contains supplementary material, which is available to authorized users.

## Background

HIV infection is a life-threatening sexually transmitted disease with rapid spread and deep impact. With approximately 36.7 million people living with HIV worldwide and an additional 2.1 million people newly infected with HIV globally in 2015, HIV/AIDS is recognized as one of the world’s worst pandemics [1]. Women account for approximately 47% of all HIV infections and 50% of new HIV infections worldwide [1].

Heterosexual intercourse remains the primary transmission route for HIV infections in women [1]. If pregnancy occurs, there is a significant concomitant risk for vertical transmission in HIV-infected women, particularly those who are not on antiretroviral therapies [2]. The use of safe and effective contraceptive methods, including hormonal contraception, is a cost-effective global health strategy with profound personal, familial and societal benefits, including the prevention of unintended pregnancies, reduced maternal and child morbidity and mortality, and decreased perinatal transmission of HIV; these, in turn, slow population growth and promote economic development [3–6]. Hormonal contraception is generally safe, acceptable, effective and used by 100 million women worldwide [7]. Long-acting contraceptive methods include injectables, implants, hormone-releasing intrauterine systems and vaginal rings. Medroxyprogesterone acetate (MPA), administered for contraception as Depo-MPA (DMPA) or Depo-Provera, is a 150 mg three-monthly intramuscular injection [8]. DMPA is one of the most commonly used hormonal contraceptive methods in the world, particularly in Sub-Saharan Africa, and the only injectable contraceptive currently available in the United States [9]. The levonorgestrel-releasing intrauterine device (LNG-IUD) containing 52 mg LNG is a highly effective, progestin-only, long-acting and reversible contraceptive method that is regaining popularity in the United States [10]. The NuvaRing, an etonogestrel (ETG)-delivering vaginal ring used over a 4-week cycle, is an effective and well-tolerated method of contraception that releases 15 μg of ethinyl estradiol and 120 μg of ETG daily [11].

Despite excellent efficacy and wide global uptake, hormonal contraception, does not provide protection against sexually transmitted diseases. Moreover, a number of human epidemiological studies and meta-analyses have suggested that hormonal contraception, including oral contraceptive pills and injectable progestin-only contraceptives (e.g., DMPA), may increase the risk of HIV acquisition, transmission and disease progression [12–19]. Other studies have failed to demonstrate an association between hormonal contraception and HIV acquisition and disease progression [20–23]. It has been demonstrated that pregnancy, a state of high and sustained progesterone levels, increases the shedding of HIV in the female genital tract [24] and is associated with elevated risk of HIV acquisition [25]. This relationship is based on epidemiological and laboratory data. Yet, uncertainty regarding the effects of hormonal contraception on a woman’s risk of HIV acquisition remains. Because heterosexual intercourse is the main transmission route of HIV infection in women, elucidating the interaction between use of hormonal contraception and female reproductive tract susceptibility to HIV is critical.

HIV uses the membrane receptor CD4 in combination with chemokine co-receptors to gain entry into host cells [26, 27]. The most important co-receptors in HIV pathogenesis are CXCR4 and CCR5 [26–28]. We hypothesized that progestin-containing contraceptives may change the expression of HIV co-receptors on immune cell targets and thus facilitate HIV recruitment and transmission. In our study, we investigated the effects of three long acting reversible contraceptives on the expression of CXCR4 and CCR5 on CD4^+^ and CD8^+^ T cells in the female genital tract and peripheral blood. Three hormonal contraceptive methods were studied and compared: DMPA, the LNG-IUD and the ETG-delivering vaginal ring. We found that administration of DMPA increased the proportion of CD4^+^ and CD8^+^ T cells expressing CCR5 in the cervix but not in peripheral blood. This effect on cervical HIV target cells was not noted with the LNG-IUD or ETG vaginal ring. Our findings provide at least one plausible biological explanation for the association between DMPA use and elevated risk of heterosexual HIV transmission.

## Methods

### Study subjects

The experimental procedures were performed between March 2014 and July 2016. A total of 59 healthy women aged 18 to 30 who attended the General Obstetrics and Gynecology Clinic at the University of Missouri, Columbia, USA or the Department of Obstetrics and Gynecology of Guangzhou First People’s Hospital, China and requested long-acting progestin-containing contraception completed both study visits and were therefore included in our data analysis. Fifteen patients chose DMPA and 150 mg of the drug was given intramuscularly at the time of study entry; 28 chose the LNG-IUD (Mirena, Bayer Pharmaceuticals, Berlin, Germany), which was inserted under sterile conditions at the initial study visit; and 16 chose the ETG vaginal ring (NuvaRing, Merck & Co., Kenilworth, NJ), which was inserted by patient or provider with ring placement verified by the provider after sample collection at the first study visit.

Subjects within 7 days of their last menstrual period (early follicular phase) or at their 6–7 week post-partum were recruited. Post-partum women were not lactating and had not returned to normal menses. Enrollees were instructed to schedule a second visit to clinic 3–4 weeks after initiation of contraception. ETG vaginal ring users were specifically instructed to return for their visit three weeks after ring insertion but before removal of the device. Subjects were asked to refrain from intercourse and douching for 48 h prior to each visit. Face-to-face interviews were conducted to gather information on demographics and sexual behavioral parameters. We included women who were healthy, HIV-negative, non-pregnant and had regular menstrual cycles. We excluded individuals who 1) used any hormonal contraception within 60 days of enrollment; 2) were pregnant or less than 6 weeks from delivery; 3) were diagnosed with precancerous lesions, carcinoma, trauma or cervical dysplasia; 4) underwent any genital tract procedure including biopsy; 5) had a new sexual partner within 3 months of enrollment; 6) used any steroids within 1 year of enrollment; 7) had any contraindication to IUD or vaginal ring use or 8) had signs on exam or symptoms of active genital tract infection.

### Sample collection

Peripheral blood and endocervical cytobrush specimens were collected to analyze HIV target cell populations at both visits (2 total collections). Peripheral blood was collected by venipuncture using heparin as an anticoagulant to isolate peripheral blood mononuclear cells (PBMCs) and for HIV RNA testing using the Abbott RealTime HIV-1 Assay (Abbott Molecular, Des Plaines, IL). Endocervical specimens were obtained to isolate endocervical cells by inserting a cytobrush into the cervical os, rotating 360° and placing the cytobrush in 1 ml of RPMI-1640 (Life Technologies, Grand Island, NY) [29]. Posterior fornix swabs were obtained to detect the presence of bacterial vaginosis and sexually transmitted diseases. One vaginal swab was obtained for assessing bacterial vaginosis, *Candida* species and *Trichomonas vaginalis* using Gram staining and direct visualization. One vaginal swab was collected at the initial entry visit to determine the presence of *Chlamydia trachomatis* using nucleic acid amplification testing (NAAT, Gen-Probe, San Diego, CA). Another vaginal swab was collected to detect the presence *Neisseria gonorrhoeae* using direct culture.

### Sample processing

Endocervical cytobrush specimens and blood were collected prior to administration of contraception and processed within 1 h of collection. Cytobrushes were agitated gently in the collection fluid and washed with RPMI-1640 several times to remove as many cells as possible. Cell suspensions were then filtered through a 40-μm cell strainer (Thermo Fisher Scientific, Waltham, MA). After filtering, cells were washed and re-suspended in 50 μl PBS for flow cytometry. PBMCs were isolated over a Ficoll-Paque Premium (GE Healthcare, Pittsburgh, PA) by centrifugation at 400×g for 30 min at 20 °C and re-suspended in 50 μl PBS for flow cytometry. Cell viability for both endocervical cells and PBMCs was over 95% as judged by Trypan blue (Sigma-Aldrich, St. Louis, MO) exclusion. All laboratory personnel were blinded to clinical status of participants including hormonal contraception choice.

### Flow cytometry

Cells were incubated with allophycocyanin (APC)/Cy7-conjugated anti-CD3 Ab (SK7, 5 μl), fluorescein isothiocyanate (FITC)-conjugated anti-CD4 Ab (RPA-T4, 20 μl), APC-conjugated anti-CD8 Ab (RPA-T8, 20 μl), phycoerythrin (PE)-conjugated anti-CXCR4 Ab (12G5, 20 μl) and PE/Cy7-conjugated anti-CCR5 Ab (2D7/CCR5, 5 μl) for 30 min at 4 °C. After washing twice with PBS, cells were fixed in a fixation buffer. Isotype controls were established using matched fluorescence-labeled isotype control Abs to account for nonspecific staining. Immunostained cells were analyzed on a CyAn ADP flow cytometer (Beckman Coulter, Brea, CA) or a FACSCanto flow cytometer (BD Biosciences, San Jose, CA) using FlowJo software (Tree Star, Ashland, OR). The expression of CD4 and CD8 on CD3^+^ cells and the expression of CXCR4 and CCR5 on CD4^+^CD3^+^ and CD8^+^CD3^+^ cells were measured. Fluorescence-conjugated Abs, matched fluorescence-labeled isotype control Abs and the fixation buffer were all purchased from BD Biosciences. Although experiments were performed in two different laboratories, the laser alignment of the two flow cytometers were identical, experiments were all performed by trained postdoctoral fellows using standard protocols, and appropriate isotype antibodies were used to exclude background noise. Gating choices were overseen by the same personnel at each site.

### Statistical analysis

All statistical analyses were performed using SPSS 23.0 software (IBM, Armonk, NY, USA). Continuous variables were summarized using medians and interquartile ranges. Categorical variables were summarized using percentages and frequencies. For continuous variables, Wilcoxon testing was used for comparisons between two groups and Kruskal-Wallis H testing for multiple group comparisons. Fisher’s exact testing was used to compare categorical variables. A *p* value of < 0.05 was considered significant.

## Results

### Participant demographic data and sexual behaviors

Between March 2014 and July 2016, 83 HIV-negative women were enrolled in the study and 59 women completed both study visits including 31 from the United States and 28 from China. All subjects were tested for *Chlamydia trachomatis*, *Neisseria gonorrhoeae*, *Trichomonas vaginalis* and bacterial vaginosis. One subject was cultured positive for *Chlamydia trachomatis* and one patient tested positive for bacterial vaginosis at their initial visits. Each was treated and repeat tests were negative for *Chlamydia trachomatis* and bacterial vaginosis, respectively. Since enrollment was difficult and cases treated to cure now met inclusion criteria, these patients were included in the analyses after they were cured. These two women were both treated with DMPA and the flow cytometry results of them were shown in Additional file 1: Figure S1. No significant differences with regard to age, race, recent coitus, and condom use were observed among various contraceptive method groups (Table 1).

**Table 1:** Demographic and behavioral characteristics at enrollment^a^

	Total	DMPA	LNG-IUD	ETG vaginal ring	*p* value
Age	23.0 (20.0–26.0)	21.0 (19.0–25.0)	25.0 (20.3–27.0)	24.0 (19.0–27.5)	0.313
Race					0.784
White	23/59 (39.0%)	6/15 (40.0%)	12/28 (42.9%)	5/16 (31.3%)	
Black	6/59 (10.2%)	1/15 (6.7%)	4/28 (14.3%)	1/16 (6.3%)	
Asian	30/59 (50.8%)	8/15 (53.3%)	12/28 (42.9%)	10/16 (62.5%)	
Sexual frequency per week^b^					0.789
0	11/59 (18.6%)	3/15 (20.0%)	5/28 (17.9%)	3/16 (18.8%)	
1	23/59 (39.0%)	4/15 (26.7%)	13/28 (46.4%)	6/16 (37.5%)	
≥ 2	25/59 (42.4%)	8/15 (53.3%)	10/28 (35.7%)	7/16 (43.8%)	
Multiple sex partners^c^					0.806
Yes	9/59 (15.3%)	3/15 (20.0%)	4/28 (14.3%)	2/16 (12.5%)	
No	50/59 (84.7%)	12/15 (80.0%)	24/28 (85.7%)	14/16 (87.5%)	
Condom use					0.698
Always	11/59 (18.6%)	4/15 (26.7%)	5/28 (17.9%)	2/16 (12.5%)	
Sometimes	25/59 (42.4%)	7/15 (46.7%)	10/28 (35.7%)	8/16 (50.0%)	
Never	23/59 (39.0%)	4/15 (26.7%)	13/28 (46.4%)	6/16 (37.5%)	

^a^Data were analyzed using Fisher’s exact text for categorical data and Kruskal-Wallis H test for continuous data across three groups, and are presented as median and interquartile range or n/N (%)

^b^This variable was analyzed in the past 30 days

^c^This variable was measured in the past 3 months

### Effects of DMPA on the percentages of CXCR4^+^ and CCR5^+^ cells among peripheral blood and endocervical T cells

**Fig. 1:**
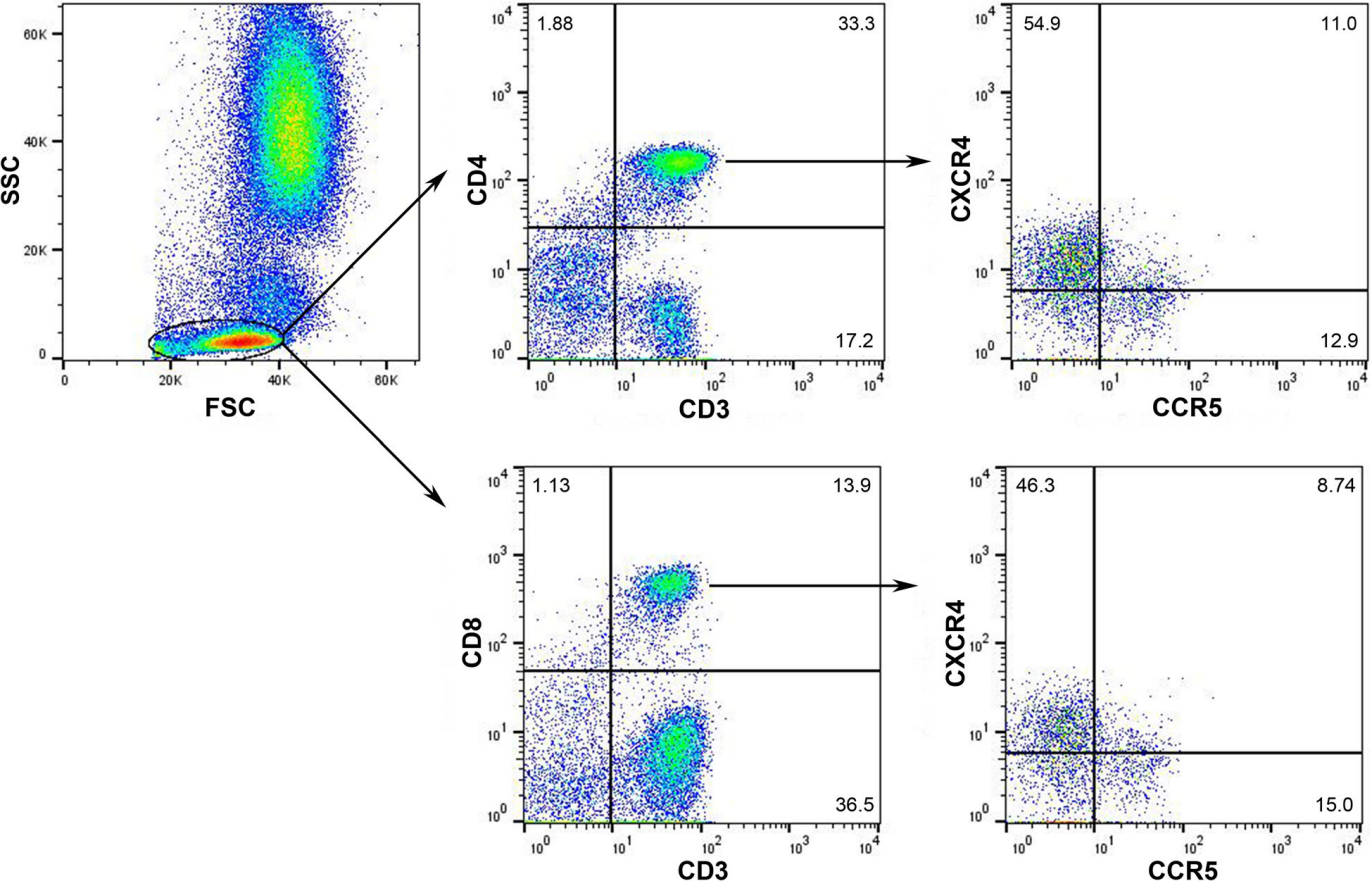
PBMC gating strategy. PBMCs were isolated as described. An initial acquisition gate on the FSC versus the SSC profile that excluded most of the non-lymphocytes was introduced to minimize background fluorescence. T cells were identified using an anti-CD3 Ab. CD4^+^CD3^+^ cells and CD8^+^CD3^+^ cells were then gated and screened for the expression of the chemokine receptors CXCR4 and CCR5

A total of 10^5^ PBMCs were analyzed. An initial acquisition gate on the forward light scatter (FSC) versus the side light scatter (SSC) profile that excluded most of the non-lymphocytes was introduced to minimize background fluorescence. T cells were identified using an anti-CD3 mAb. CD4^+^CD3^+^ cells and CD8^+^CD3^+^ cells were then gated and screened for the expression of the chemokine receptors CXCR4 and CCR5. Our gating strategy for PBMCs is shown in Fig. 1. Exposure to DMPA treatment did not affect the percentages of peripheral blood CD4^+^ or CD8^+^ T cells that expressed CXCR4 or CCR5 (Fig. 3a).

**Fig. 2:**
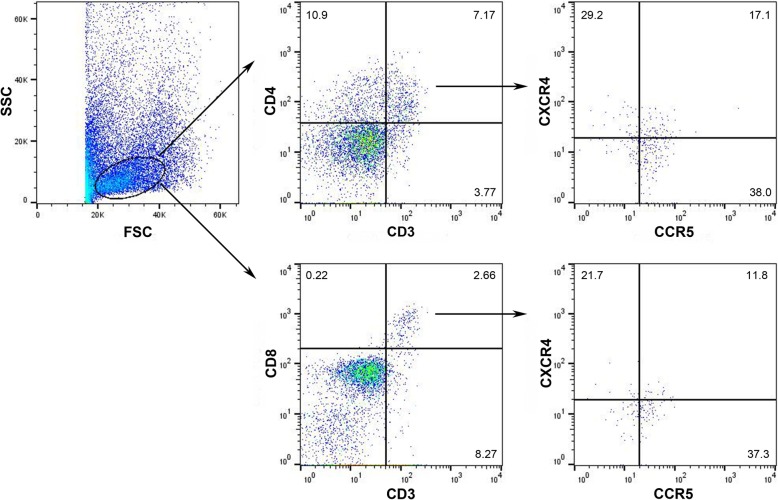
Endocervical cell gating strategy. Endocervical cells were isolated as described. An initial acquisition gate on the FSC versus SSC profile that excluded most of the non-lymphocytes was introduced to minimize background fluorescence. T cells were identified using an anti-CD3 Ab. CD4^+^CD3^+^ cells and CD8^+^CD3^+^ cells were then gated and screened for the expression of the chemokine receptors CXCR4 and CCR5

**Fig. 3:**
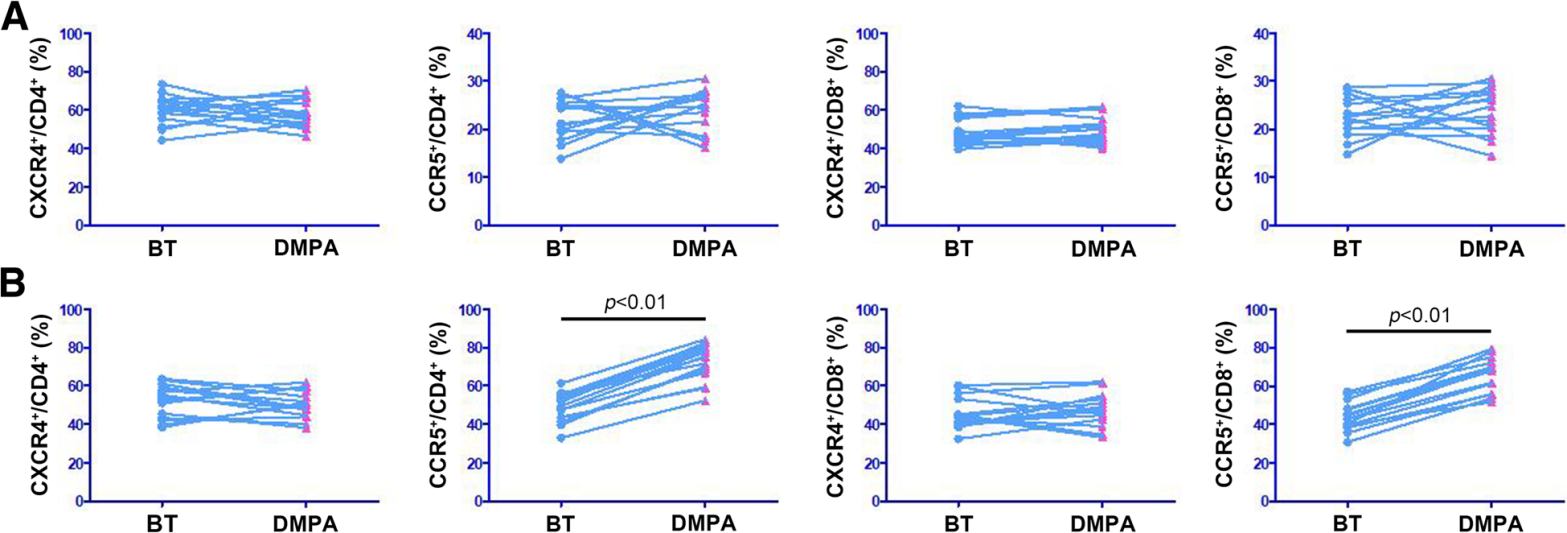
Effects of DMPA on the percentages of CXCR4^+^ and CCR5^+^ cells in peripheral blood and endocervical T cells. Women were treated with DMPA as described. PBMCs and endocervical cells were isolated at enrollment and approximately 3–4 weeks later. The percentages of CXCR4^+^ cells and CCR5^+^ cells in peripheral blood (**a**) and endocervical CD4^+^ and CD8^+^ T cells (**b**) were analyzed using flow cytometry. Data were analyzed using Wilcoxon testing and are presented as paired samples for each individual patient before and after initiation of contraception (*n* = 15). BT: before treatment

In light of data suggesting that the endocervical mucosa is an important portal for HIV acquisition [30], we assessed the frequency of CXCR4 and CCR5 expression in endocervical CD4^+^ and CD8^+^ T cells. Our gating strategy for endocervical cell specimens is shown in Fig. 2. The number (median and interquartile range) of CD3^+^ endocervical T cells was 739 (548–914). DMPA treatment significantly increased the percentage of CD4^+^ endocervical T cells that expressed CCR5 from about 48.7 to 72.3% (*p* < 0.01) (Fig. 3b). To highlight the ability of our study design to use subjects as their own internal control pre- and post-initiation of long-active progestin containing contraception, we have here presented our data in plots that allow per patient linkage to be shown. We used standard plots for all other comparisons since no hormone exposure effects were detected. The frequency of CCR5^+^ cells among endocervical CD8^+^ T cells was also significantly increased in women using DMPA (66.1%) when compared to levels before treatment (44.0%) (*p* < 0.01) (Fig. 3b). We identified no differences in the frequencies of CXCR4^+^ cells among cervical CD4^+^ and CD8^+^ T cells (Fig. 3b).

### Effects of the LNG-IUD on the percentages of CXCR4^+^ and CCR5^+^ cells among peripheral blood and endocervical T cells

**Fig. 4:**
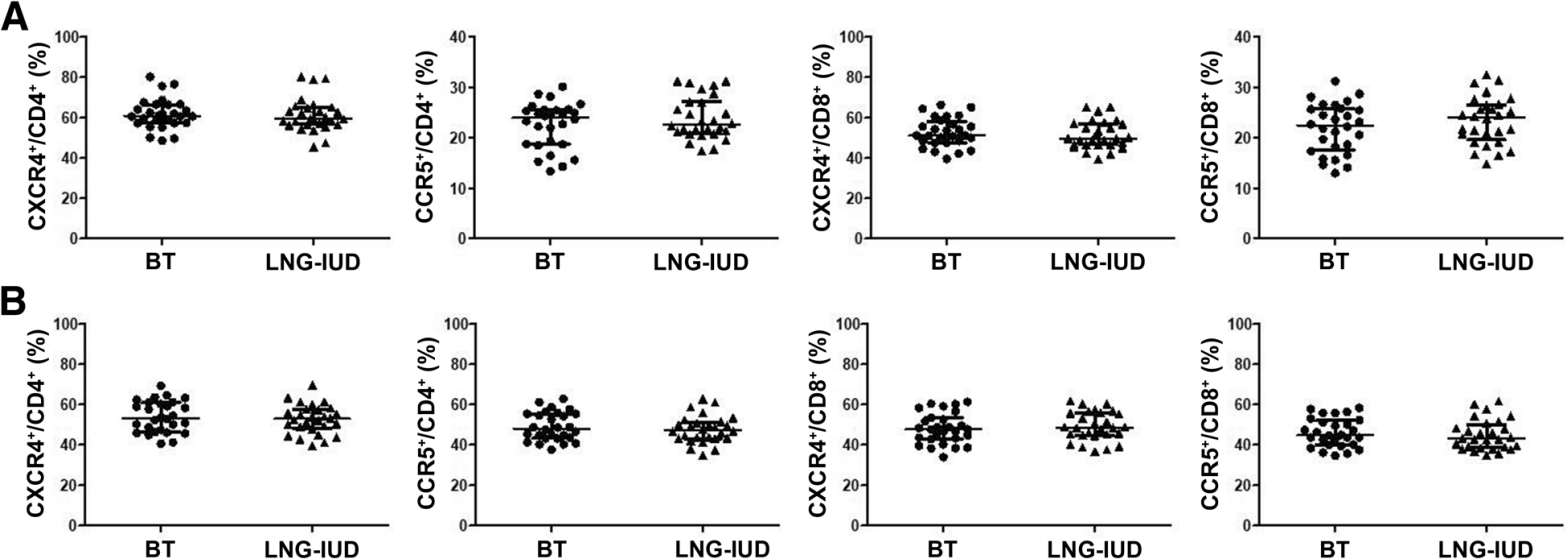
Effects of the LNG-IUD on the percentages of CXCR4^+^ and CCR5^+^ cells in peripheral blood and endocervical T cells. Women were treated with the LNG-IUD as described. PBMCs and endocervical cells were isolated at enrollment and approximately 3–4 weeks later. The percentages of CXCR4^+^ cells and CCR5^+^ cells in peripheral blood (**a**) and endocervical CD4^+^ and CD8^+^ T cells (**b**) were analyzed using flow cytometry. Data were analyzed using Wilcoxon testing and are presented as median and interquartile range (*n* = 28). BT: before treatment

No significant differences were detected in the percentages of CXCR4^+^ or CCR5^+^ cells in peripheral blood T cells between before treatment and post-exposure follow-up in women initiating the LNG-IUD (Fig. 4a). LNG-IUD exposure also did not affect the frequencies of CXCR4 or CCR5 expression in endocervical CD4^+^ T cells or CD8^+^ T cells (Fig. 4b).

### Effects of the ETG vaginal ring on the frequencies of CXCR4 and CCR5 expression in peripheral blood and endocervical T cells

**Fig. 5:**
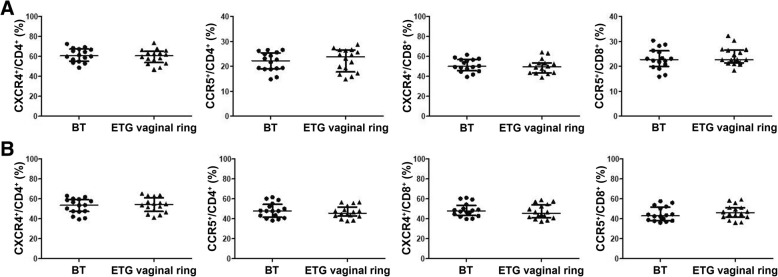
Effects of the ETG vaginal ring on the percentages of CXCR4^+^ and CCR5^+^ cells in peripheral blood and endocervical T cells. Women were treated with the ETG vaginal ring as described. PBMCs and endocervical cells were isolated at enrollment and approximately 3–4 weeks later. The percentages of CXCR4^+^ cells and CCR5^+^ cells in peripheral blood (**a**) and endocervical CD4^+^ and CD8^+^ T cells (**b**) were analyzed using flow cytometry. Data were analyzed using Wilcoxon testing and are presented as median and interquartile range (*n* = 16). BT: before treatment

We identified no differences in the percentages of CD4^+^ or CD8^+^ peripheral blood T cells that expressed CXCR4 or CCR5 between before treatment and post-exposure follow-up in women initiating the ETG vaginal ring (Fig. 5a). ETG vaginal ring exposure also did not affect the frequencies of CXCR4 or CCR5 expression in endocervical CD4^+^ or CD8^+^ T cells (Fig. 5b).

### Comparison of the expression of CXCR4 and CCR5 on peripheral blood and endocervical T cells between American and Chinese women

**Fig. 6:**
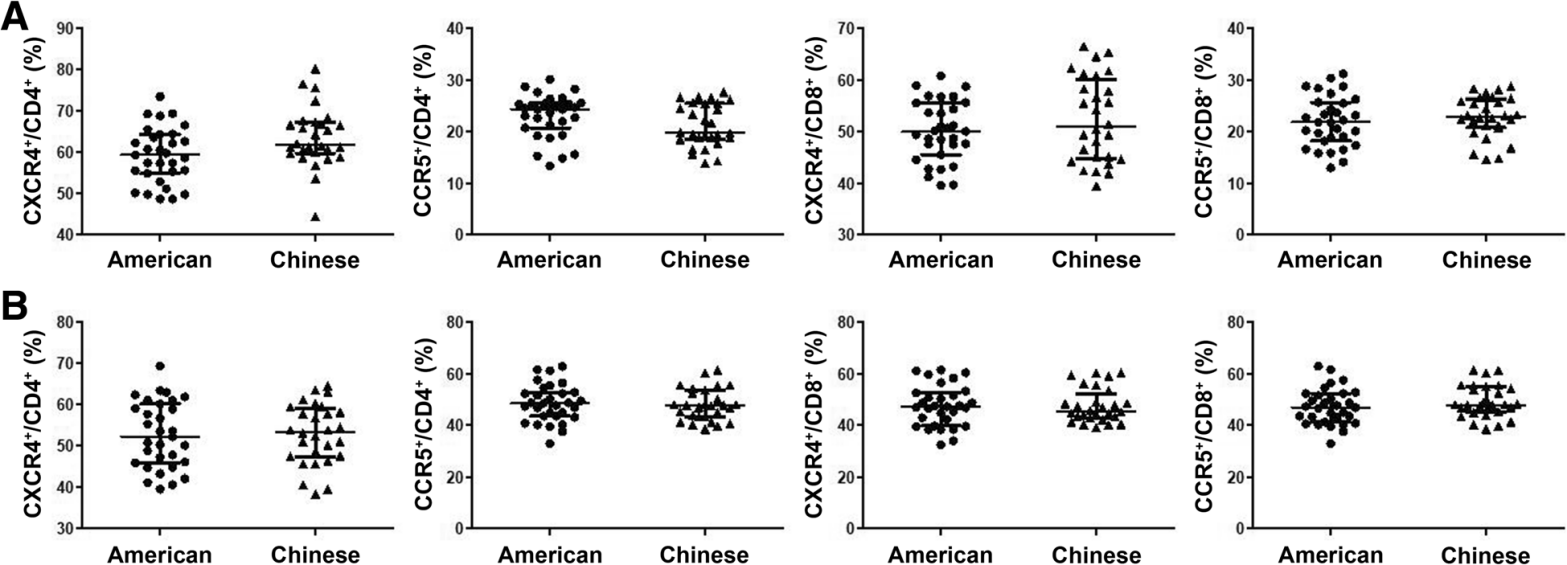
Comparison of CXCR4^+^ and CCR5^+^ T cells in peripheral blood and endocervical T cells between American and Chinese women. The percentages of CXCR4^+^ and CCR5^+^ cells in peripheral blood (**a**) and endocervical CD4^+^ and CD8^+^ T cells (**b**) in American women (*n* = 31) and Chinese women (n = 28) were analyzed at enrollment. Data were analyzed using Wilcoxon testing and are presented as median and interquartile range

Since ethnically and geographically disparate groups of women (American and Chinese) were enrolled in our study, we assessed whether these two cohorts could exhibit baseline differences in T cell expression of CXCR4 and CCR5 that might affect HIV acquisition risk. The mean and median days from pre-exposure collections to follow-up collections in American women (27.6 and 28) did not differ from those in Chinese women (25.8 and 27). The percentages of CD4^+^ and CD8^+^ T cells expressing CXCR4 and CCR5 in peripheral blood did not differ between American and Chinese women (Fig. 6a). We also identified no differences in the frequencies of endocervical CD4^+^ or CD8^+^ T cells that expressed CXCR4 or CCR5 between American and Chinese women (Fig. 6b).

### Comparison of the expression of CXCR4 and CCR5 on peripheral blood and endocervical T cells between women with normal menses and women who were post-partum

**Fig. 7:**
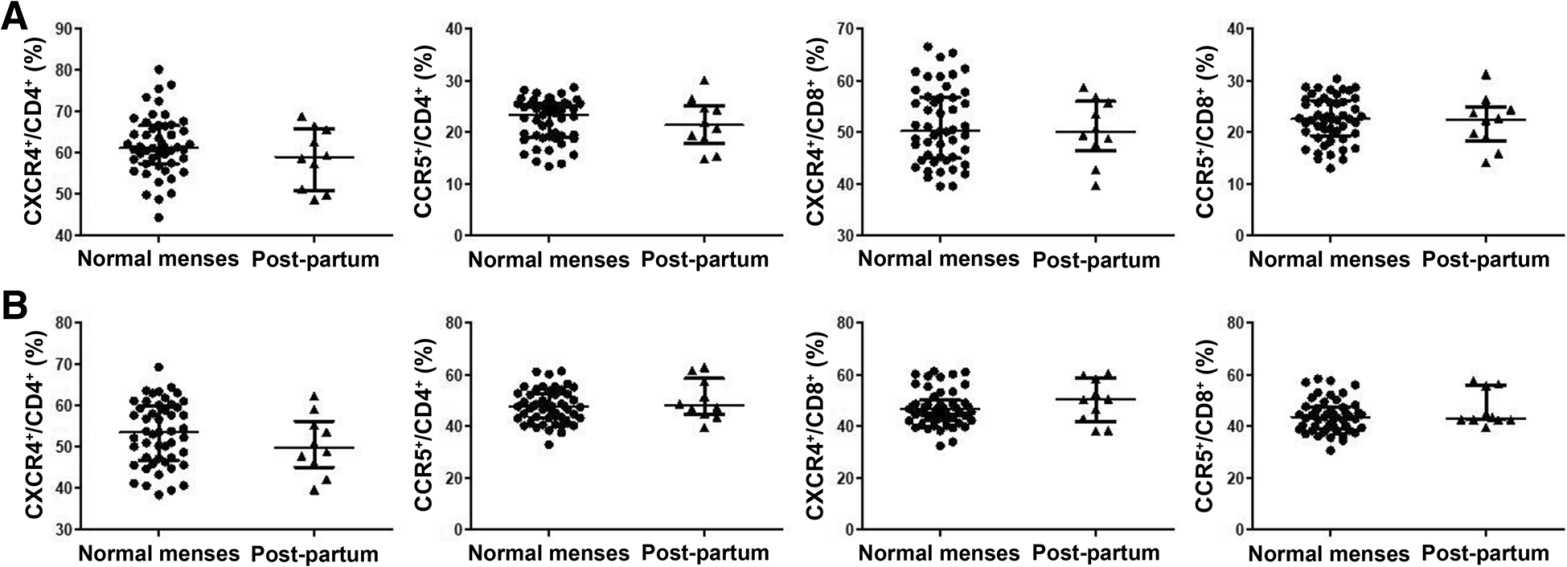
Comparison of CXCR4^+^ and CCR5^+^ T cells in peripheral blood and endocervical T cells between normally cycling and post-partum women. The percentages of CXCR4^+^ and CCR5^+^ cells in peripheral blood (**a**) and endocervical CD4^+^ and CD8^+^ T cells (**b**) in women with normal menses (*n* = 49) and post-partum women (*n* = 10) were analyzed at enrollment. Data were analyzed using Wilcoxon testing and are presented as median and interquartile range

Ten women were included in our study who were post-partum and had not yet resumed menses. We compare the percentages of peripheral blood and endocervical T cells expressing CXCR4 and CCR5 in women with normal menses and women who were post-partum to assess whether these combined analyses were appropriate. The mean and median days from pre-exposure collections to follow-up collections in normally cycling women (26.6 and 27) did not differ from those in post-partum period (27.4 and 28). No differences were detected in the percentages of CD4^+^ T cells or CD8^+^ T cells expressing CXCR4 or CCR5 in either the peripheral blood (Fig. 7a) or endocervix (Fig. 7b) in normally cycling versus post-partum women.

### Comparison of the T cell subsets in paired peripheral blood and cervical specimens

To determine whether certain characteristics of endocervical immunity may promote susceptibility to sexual transmission of HIV at this site, we compared the frequencies of T cell subsets in paired peripheral blood and endocervical specimens obtained from women before exposure to any form of hormonal contraception. As shown in Table 2, CD4^+^ T cells were the predominant T lymphocyte subset within the peripheral blood and endocervix, which is in agreement with the results reported by Prakash et al. [31]. The frequency of CD4^+^ cells among endocervical CD3^+^ cells was statistically higher than that in matched peripheral blood (*p* < 0.01). In contrast, the frequency of CD8^+^CD3^+^ cells in endocervical specimens was significantly lower than that in peripheral blood (*p* < 0.01).

**Table 2:** T cell subsets in paired peripheral blood and cervix at enrollment (%)

	CD4^+^/CD3^+^	CD8^+^/CD3^+^	CXCR4^+^/CD4^+^	CCR5^+^/CD4^+^	CXCR4^+^/CD8^+^	CCR5^+^/CD8^+^
Blood	58.9 (55.4–64.1)	32.6 (28.4–34.3)	61.2 (57.4–66.5)	23.1 (18.9–25.5)	50.2 (45.2–56.7)	22.6 (19.1–25.9)
Cervix	74.3 (65.4–78.9)^a^	18.7 (14.6-23.7)^a^	53.6 (46.3-59.6)^a^	47.9 (43.3-55.2)^a^	46.8 (42.1-53.2)^b^	43.7 (39.4-51.1)^a^

Data were analyzed using Wilcoxon test and are presented as median and interquartile range

^a^*p* < 0.01 vs. the Blood group

^b^*p* < 0.05 vs. the Blood group

Finally, the proportions of CD4^+^ and CD8^+^ T cells in the endocervix that expressed CXCR4 were significantly lower than those in peripheral blood (*p* < 0.01 and *p* < 0.05). However, there were higher frequencies of CCR5-expressing CD4^+^ and CD8^+^ T cells in endocervical mucosae when compared to matched peripheral blood (*p* < 0.01 for both comparisons). In short, there is a higher frequency of CCR5^+^ cells and lower frequency of CXCR4^+^ cells in the cervix than in peripheral blood. Both the higher proportion of potential CD4^+^ HIV target T cells and the higher frequency of CCR5 positivity in these cells in the endocervix when compared to matched peripheral blood may facilitate sexual HIV transmission.

## Discussion

There are several limitations to epidemiological studies suggesting a possible association between use of hormonal contraception and an increased risk of HIV infection. These include the observational nature of these studies, infrequent and imprecise measurement of hormonal contraception exposure and HIV acquisition, and differences in study participant sexual behaviors, particularly condom use, between users and non-users of hormonal contraception [7, 13, ﻿32﻿]. Such limitations may mask the true biological effects of hormonal contraception on HIV acquisition. Defining the mechanisms by which hormonal contraceptives alter HIV acquisition and transmission may provide insight into some of the inconsistencies in existing epidemiological data and suggest new investigative approaches and/or safer contraceptive options for targeted use in areas with high HIV prevalence and high fertility rates.

The endocervix has been proposed as an important initial site for the entry of HIV in women [30]. This epithelial barrier has a single epithelial cell layer and contains numerous CD4^+^ T cells in the luminal and glandular components as well as the lamina propria [﻿33﻿]. These CD4^+^ T cells are believed to be the primary targets in sexual transmission of HIV [34]. In this study, we analyzed the effects of progestin-containing hormonal contraceptives on the frequency of HIV target cells in the endocervical mucosa and paired peripheral blood to investigate potential immunological mechanisms underlying reported DMPA-mediated increases in the risk for HIV acquisition. By comparing to other long-acting progestin-containing contraceptive methods, we sought to begin to identify safer contraceptive alternatives for high-risk women.

Since no significant differences in the baseline frequencies of CXCR4 or CCR5 positivity were identified in peripheral blood or endocervical T cells between American and Chinese women or between normally cycling and post-partum women in our study, we analyzed American and Chinese women together, and cycling and post-partum women together. Since most women were in the early follicular phase at both visits and the outcomes did not differ between normally cycling and post-partum women, we think temporal changes contribute little to the analyses.

CCR5 is the predominant co-receptor for R5 strains of HIV. These R5-dependent viruses are responsible for the majority of sexually transmitted HIV infections [35]. CCR5 has been detected on genital tract T cells [12] and is thought to be the predominant co-receptor target for initial sexual transmission of HIV [﻿﻿33﻿]. An increase in the proportion of endocervical CD4^+^ T lymphocytes that express CCR5 in our study offers a potential mechanism for the increased risk of HIV acquisition seen in women receiving DMPA. Our data, gathered from groups of Chinese women and of mostly Caucasian women from the United States, are in accordance with the results of Byrne et al. [12], which demonstrated a significant increase in cervical CCR5-bearing lymphocytes in women at risk for HIV infection and using DMPA in South Africa. However, it contradicts the cross-sectional study of Smith-McCune et al. that enrolled mostly Black and Caucasian women and compared fairly long-term users of DMPA (> 6 mo) to a cohort of control women in the mid-luteal phase and on no hormonal contraception [36]. Discrepancies between our results and those of the latter study results may reflect study design differences, including comparison in the Smith-McCune paper to a high endogenous progesterone state in controls (mid-luteal), while our study included many women in their follicular phase before treatment. Possible, but arguably less likely are the demographic differences between our study and theirs.

The higher frequency of CCR5^+^ cells in the cervix upon DMPA exposure may reflect preferential recruitment of CCR5^+^ cells to the site although increased CCR5 expression on existing cells may also be involved. In fact, an in vitro study has demonstrated that CCR5 expression in explanted cervical tissue is enhanced by exogenous progesterone [﻿32﻿]. In addition, both effects of DMPA on CCR5 have been reported to be associated with elevated CCR5-bearing immune cells in female vaginal tissue [9]. Elevation in the frequency of endocervical CD8^+^ T cells expressing CCR5 could also facilitate HIV binding and promote cell apoptosis; such mechanisms need to be further investigated.

CXCR4 is the main co-receptor for X4 strains of HIV which are primarily responsible for disease progression, although they can be associated with primary transmission [37]. In this study, we did not detect significant changes in the proportion of endocervical T cells that expressed CXCR4 upon DMPA treatment. Nor could we link the use of DMPA to changes in the proportion of peripheral blood T cells expressing CXCR4 or CCR5. Our results indicate that DMPA exposure has a preferential and specific effect on CCR5^+^ endocervical T cells in a manner that would be predicted to facilitate sexual transmission of HIV across the female genital tract.

There are limited data on the effects of the LNG-IUD on the immune cell subsets in the female genital tract. In our study, use of the LNG-IUD did not increase the frequency of CXCR4 or CCR5 positive T cells in the endocervix; these frequencies were unchanged before and after exposure to the device. Achilles et al. [10] reported decreased CCR5 expression on CD4^+^ T cells in the cervix and endometrium after 2 months use of the LNG-IUD when compared with pre-insertion levels in a cohort of mostly Caucasian women. Differences between the results of our study and that of Achilles et al. may be a function of population differences and/or hormone exposure time; the latter will be addressed in an ongoing study.

In contrast, Sciaranghella et al. [﻿38﻿] showed that use of LNG-IUD was associated with increased CCR5 expression on peripheral blood T cells in mostly Black and Hispanic HIV-uninfected women. In this case-control study, similar but less marked changes in CCR5 expression levels were noted in DMPA users. This relationship was not related to hormone levels and some have hypothesized it may be related to the presence of the IUD string as a foreign body. Time of use of a given contraceptive choice was not delineated in the study. Inconsistences between the results of our study and theirs may be due to the discrepancies in the study design, method of selection of study subjects, subject sample size and analytic methods used.

To our knowledge, there are no data on the effects of contraceptive vaginal rings on the expression of HIV co-receptors in the female genital tract or peripheral blood. Here, we showed that, similar to the LNG-IUD, use of the ETG vaginal ring had no effect on the proportion of CXCR4^+^ or CCR5^+^ peripheral blood or endocervical T cells. In contrast to progesterone, estrogen has been reported to thicken the vaginal epithelial layer of macaques [39] and to provide efficient protection against the transmission of SIV [40]. The ethinyl estradiol in the NuvaRing may have contributed to the unaltered expression of CXCR4 and CCR5 on T cells in peripheral blood and endocervix of exposed women.

Finally, we found that there were greater percentages of CD4^+^ cells and CCR5-expressing CD4^+^ and CD8^+^ T cells in the cervix compared with those in the peripheral blood, which may facilitate sexual transmission of R5 viruses in the cervix. These results concur with other studies [31]. We also reported a higher proportion of CCR5^+^CD4^+^ T cells in peripheral blood than that in a prior report [12]. The reasons for this discrepancy are unclear and since gating strategies in the prior report are not presented, it is difficult to exclude differences in flow cytometry analyses, which may be the most likely explanation.

There were several strengths of our study. First, the study design allowed for individual patient comparisons before and after initiation of hormonal contraception. The patient could serve as her own control. This is an important design strength that is seldom used in the existing literature. Second, we are the first to simultaneously compare three different long-acting synthetic progestin formulations and three different delivery methods, including a hormonal intrauterine device and a vaginal ring, on the HIV transmission-related phenotypes of paired endocervical and peripheral blood T cells. Third, the subjects in our study include Caucasian, Asian and African-American women from the United States and China without HIV infection. This allows the results to be quite generalizable when compared to those that include exclusively high risk populations or those with minimal ethnographic diversity.

There are several important limitations in our study as well. We did not include a control group of normal women not using any hormonal contraception or those using a non-hormonal IUD. Also, the NuvaRing releases a combination of estrogen and progestin, so an unbiased comparison of LNG and DMPA to ETG cannot be gleaned from our data. Finally, despite study design, there is heterogeneity in the menstrual cycle timing at the time of study enrollment and several patients were postpartum at the time of enrollment. Data included in Fig. 7, however, suggest that these latter factors did not significantly alter outcomes.

## Conclusions

Our demonstration of an increase in the frequency of in HIV target cells in the endocervix in vivo, solely upon DMPA administration, is in agreement with much of the rather heterogeneous studies in the existing literature and provides a plausible biological explanation for the increased risk of HIV acquisition in women using DMPA. We could not detect significant effects of the LNG-IUD or the ETG vaginal ring on HIV co-receptor expressing T cells in female cervix or in the peripheral blood. In view of the high contraceptive efficacies and low side effect profiles of the LNG-IUD and the ETG vaginal ring, they may be appropriate contraceptive substitutes for DMPA, particularly in regions of the world where HIV transmission is the highest.

## Additional file

Additional file 1: **Figure S1.** Frequencies of CXCR4^+^ and CCR5^+^ T cells in peripheral blood and endocervical T cells in the two subjects with prior infection. The percentages of CXCR4^+^ and CCR5^+^ cells in peripheral blood (**A**) and endocervical CD4^+^ and CD8^+^ T cells (**B**) in all women in the DMPA cohort (*n* = 15), including those 2 women with prior *Chlamydia trachomatis* infection or bacterial vaginosis (indicated with connecting lines) were analyzed before and after DMPA treatment. Lines connect the same cases. BT: before treatment. (DOCX 252 kb)

## Abbreviations

APC: Allophycocyanin
DMPA: Depot medroxyprogesterone acetate
ETG: Etonogestrel
FITC: Fluorescein isothiocyanate
FSC: Forward light scatter
LNG-IUD: Levonorgestrel-releasing intrauterine device
MPA: Medroxyprogesterone acetate
NAAT: Nucleic acid amplification testing
PBMCs: Peripheral blood mononuclear cells
PE: Phycoerythrin
SIV: Simian immunodeficiency virus
SSC: Side light scatter
