# Supplementary material for: Effects of three long-acting reversible contraceptive methods on HIV target cells in the human uterine cervix and peripheral blood
Source: Reprod Biol Endocrinol. 2019 Feb 22;17:26. doi: 10.1186/s12958-019-0469-8 (PMC6387540; doi:10.1186/s12958-019-0469-8)
Supplement: Supplementary file 1 — Figure S1. Frequencies of CXCR4+ and CCR5+ T cells in peripheral blood and endocervical T cells in the two subjects with prior infection. The percentages of CXCR4+ and CCR5+ cells in peripheral blood (A) and endocervical CD4+ and CD8+ T cells (B) in all women in the DMPA cohort (n = 15), including those 2 women with prior Chlamydia trachomatis infection or bacterial vaginosis (indicated with connecting lines) were analyzed before and after DMPA treatment. Lines connect the same cases. BT: before treatment. (DOCX 252 kb) [file 12958_2019_469_MOESM1_ESM.docx]

**Supplementary Material**


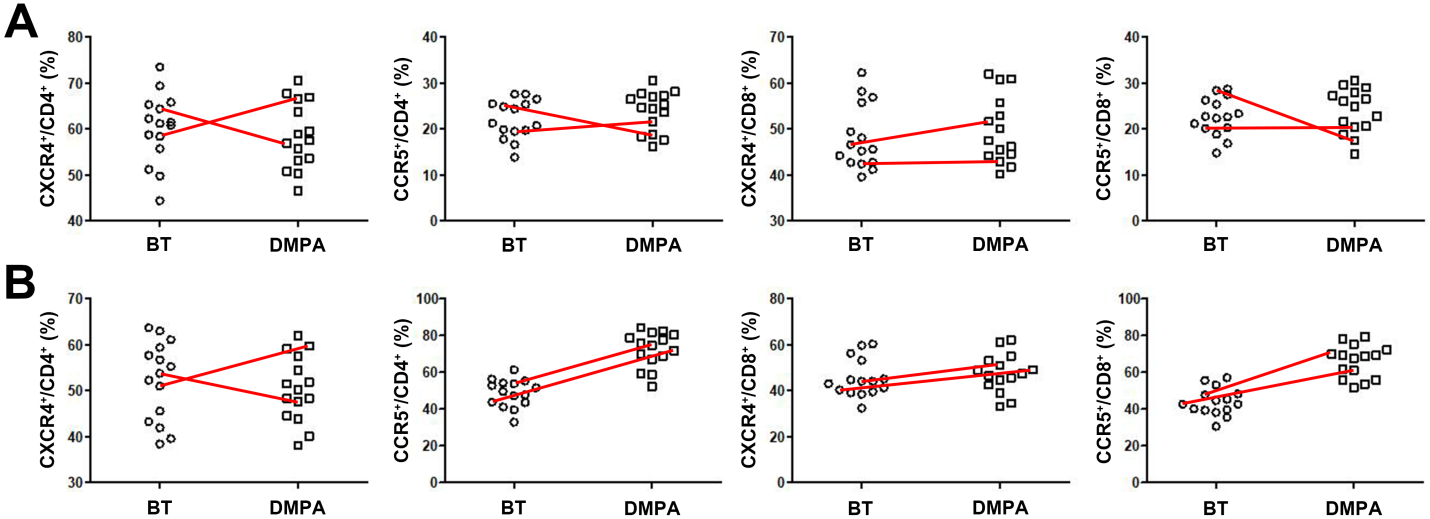


**Fig S1.** Frequencies of CXCR4^+^ and CCR5^+^ T cells in peripheral blood and endocervical T cells in the two subjects with prior infection. The percentages of CXCR4^+^ and CCR5^+^ cells in peripheral blood (**A**) and endocervical CD4^+^ and CD8^+^ T cells (**B**) in all women in the DMPA cohort (n = 15), including those 2 women with prior *Chlamydia trachomatis* infection or bacterial vaginosis (indicated with connecting lines) were analyzed before and after DMPA treatment. Lines connect the same cases. BT: before treatment.
